# Supplementary material for: Plasmonic Titanium Nitride Tubes Decorated with Ru Nanoparticles as Photo-Thermal Catalyst for CO2 Methanation
Source: Molecules. 2022 Apr 22;27(9):2701. doi: 10.3390/molecules27092701 (PMC9101908; doi:10.3390/molecules27092701)
Supplement: Supplementary file 1 [file molecules-27-02701-s001.zip › molecules-1614138-supplementary.pdf]

molecules

Supplementary Material

Plasmonic Titanium Nitride Tubes Decorated with Ru Nanoparticles as Photo-Thermal Catalyst for CO_2 Methanation

Diego Mateo *, Juan Carlos Navarro, Il Son Khan, Javier Ruiz-Martinez and Jorge Gascon *

KAUST Catalysis Center (KCC), King Abdullah University of Science and Technology (KAUST), Thuwal 23955, Saudi Arabia ; juancarlos.navarrodemiguel@kaust.edu.sa (J.C.N.); ilson.khan@kaust.edu.sa (I.S.K.); javier.ruizmartinez@kaust.edu.sa (J.R.-M.)

* Correspondence: diego.mateo@kaust.edu.sa (D.M.); jorge.gascon@kaust.edu.sa (J.G.)

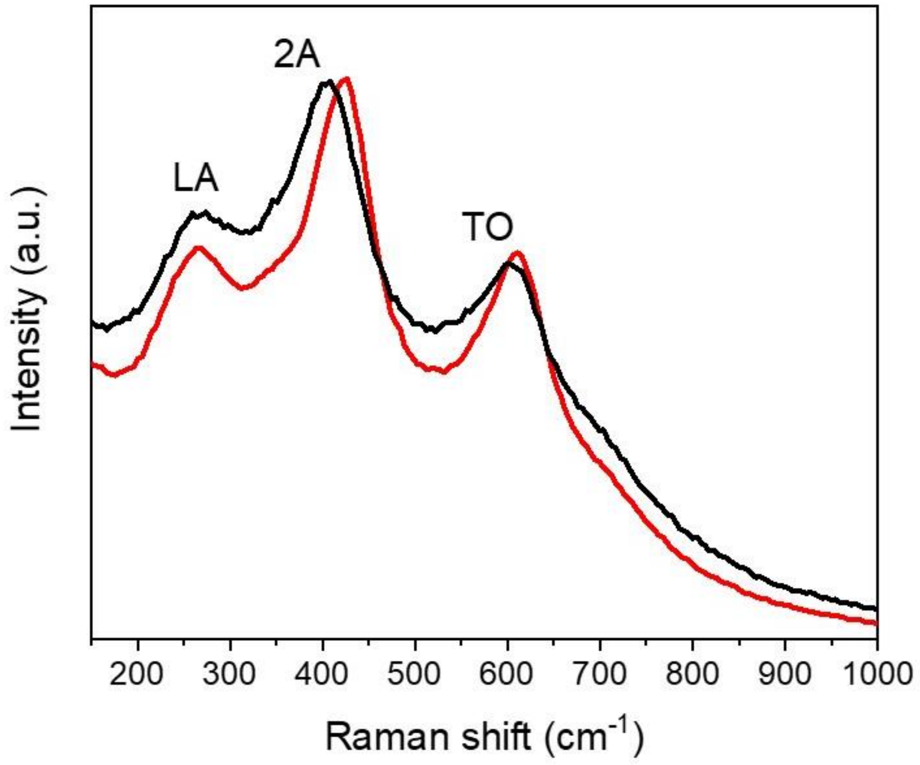

Figure S1. Raman spectra of commercial TiN (black line) and TiN tubes (red line).

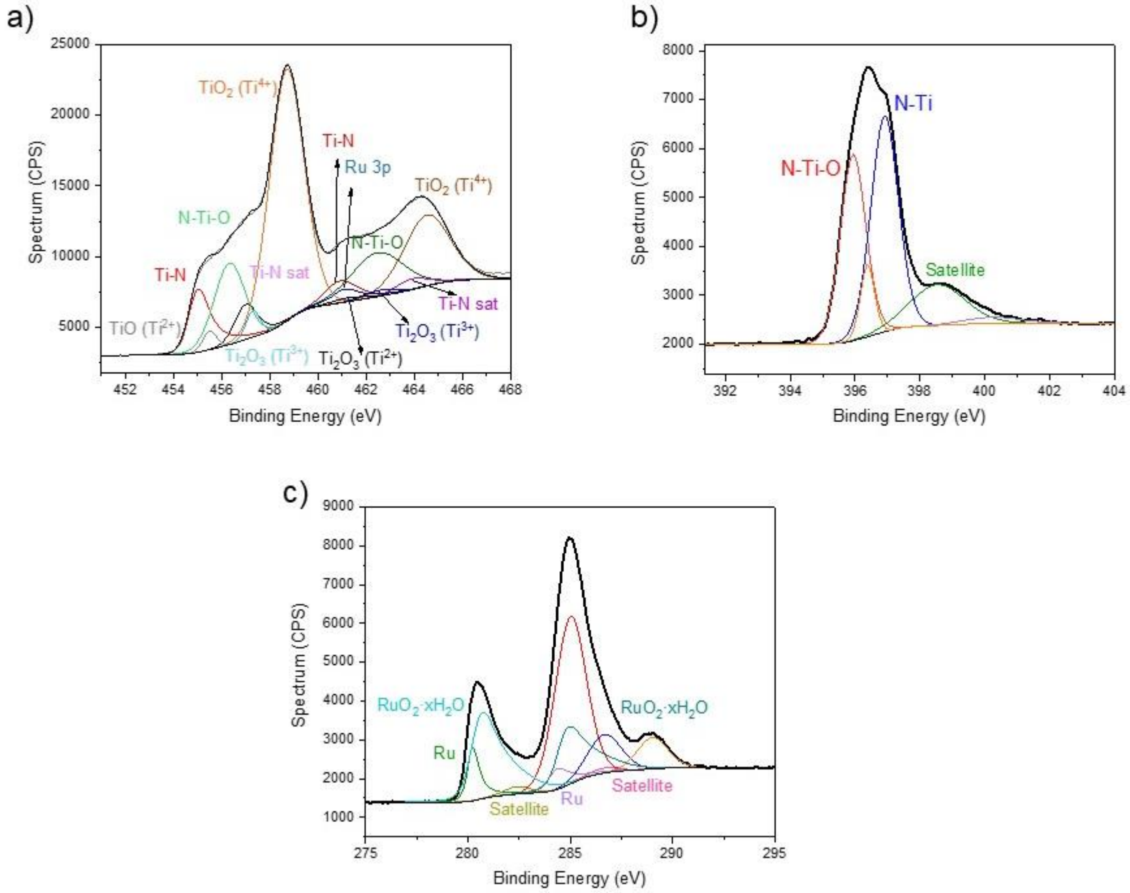

Figure S2. XPS analysis of (**a**) Ti2p, (**b**) N1s and (**c**) Ru3d regions of Ru(2)-TiN tubes photocatalyst.

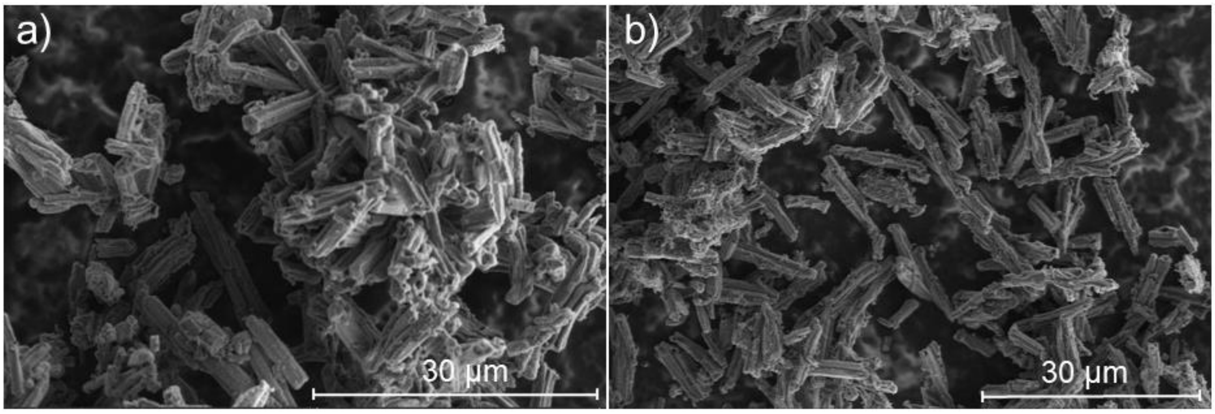

Figure S3. SEM images of (**a**) TiO_2 tubes and (**b**) TiN tubes.

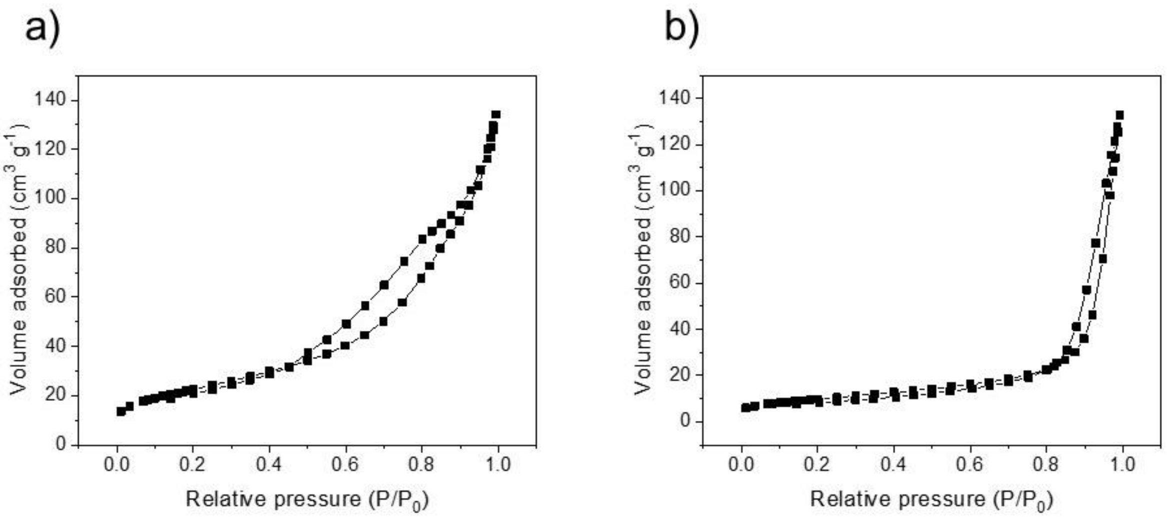

Figure S4. N_2 adsorption isotherms at 77 K. (**a**) TiO_2 tubes and (**b**) TiN tubes.

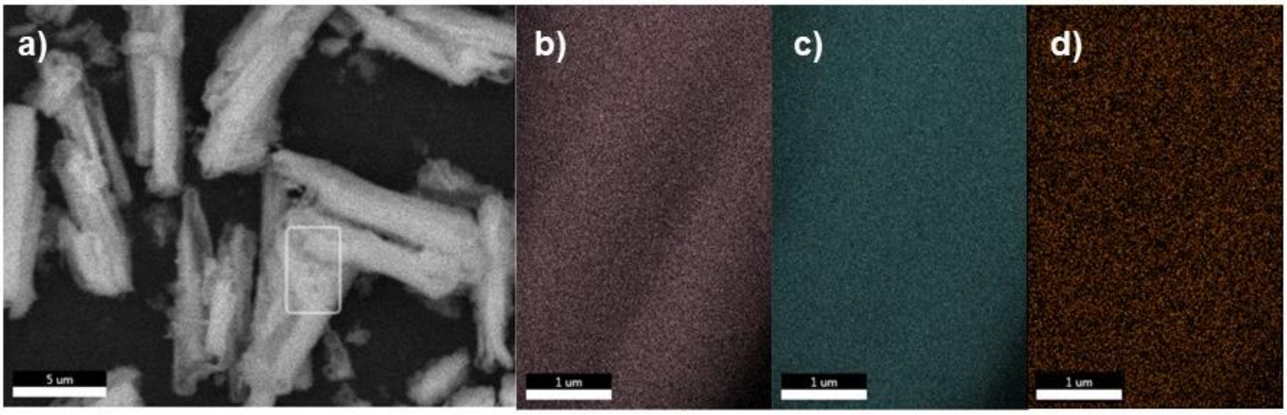

Figure S5. (**a**) SEM image of Ru(2)-TiN tubes. Elemental mapping of (**b**) N, (**c**) Ti and (**d**) Ru of selected area.

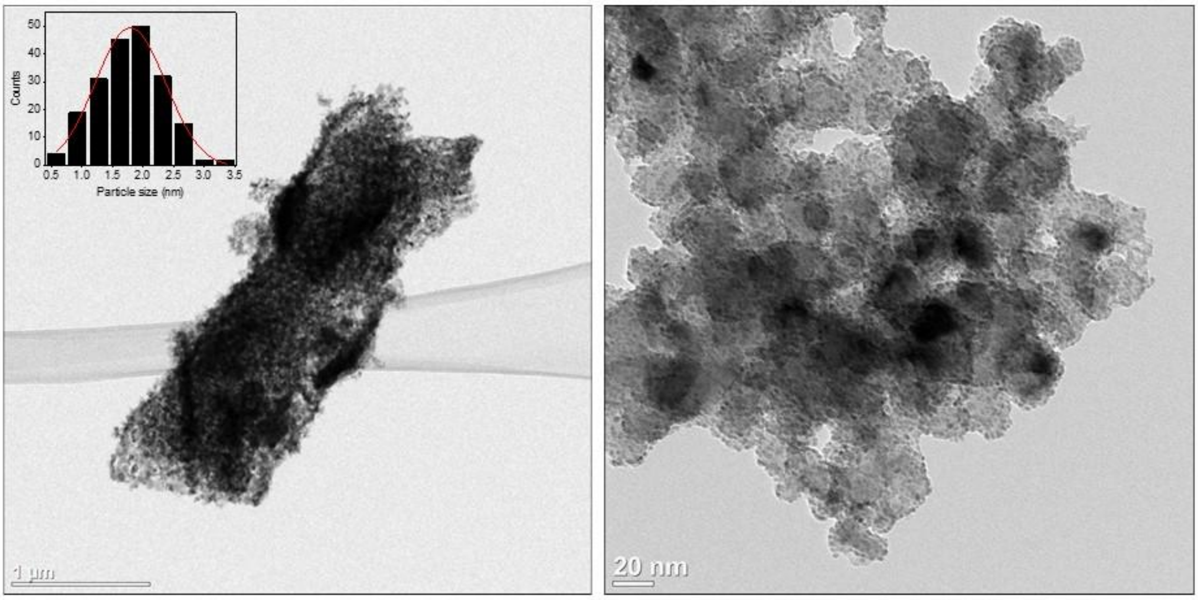

Figure S6. HRTEM images of Ru(2)-TiN tubes after reaction. Inset shows the particle size distribution.

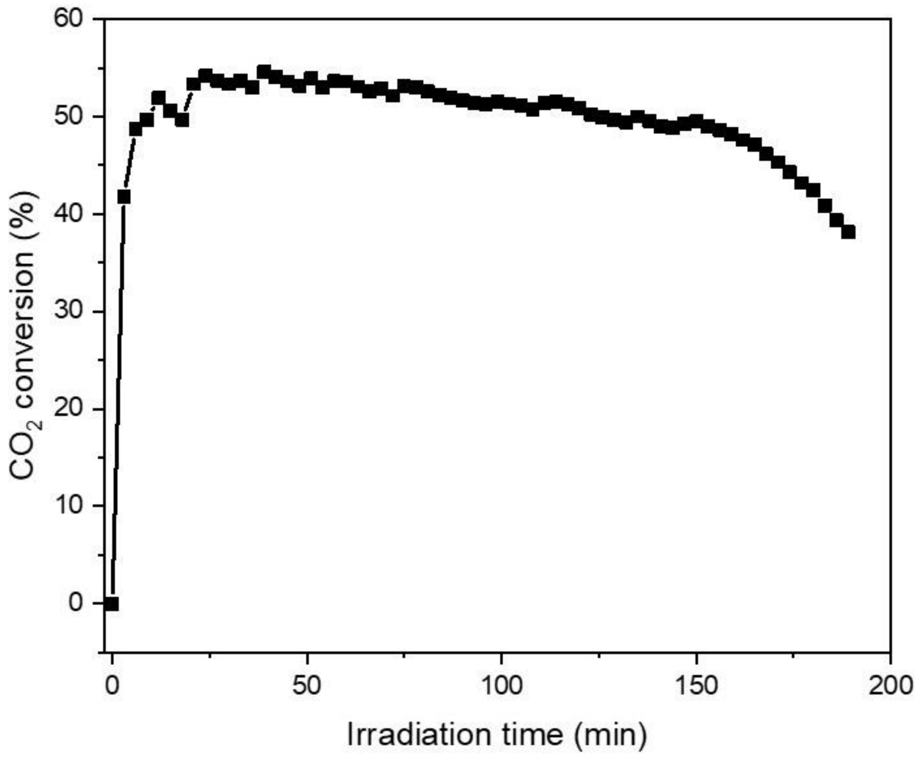

Figure S7. CO_2 conversion values of Ru(2)-TiN tubes under continuous flow configuration.

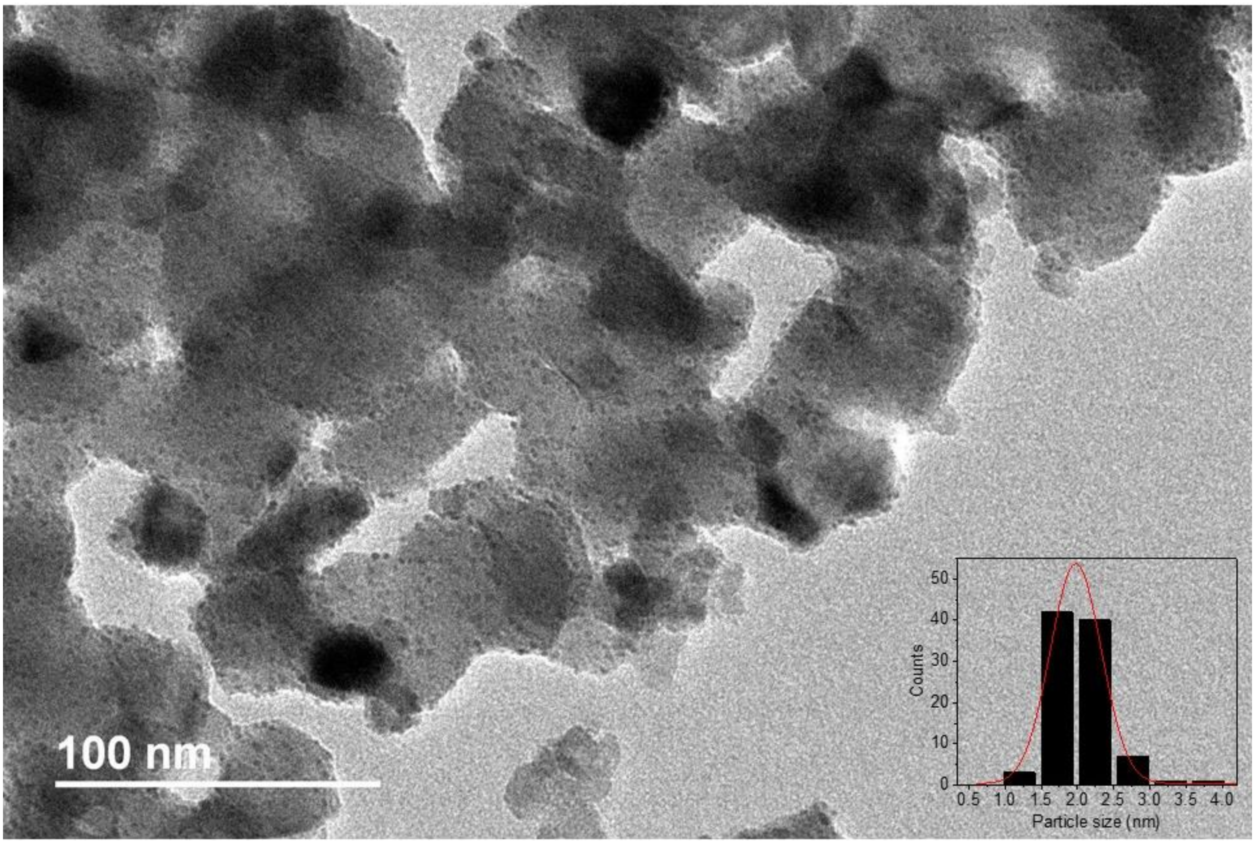

Figure S8. TEM image of Ru(2)-TiN tubes after long-term experiment under continuous flow configuration. Inset shows the particle size distribution.

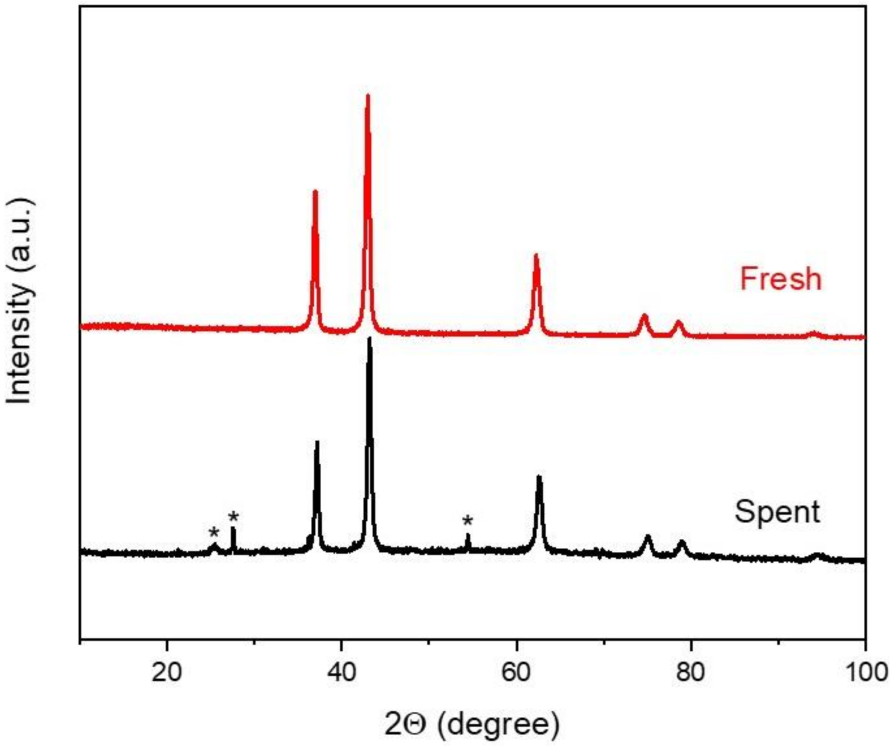

Figure S9. X-ray diffraction patterns of fresh (red) and spent (black) Ru-TiN tubes. Asterisks indicate diffraction peaks corresponding to TiO_2 phase.

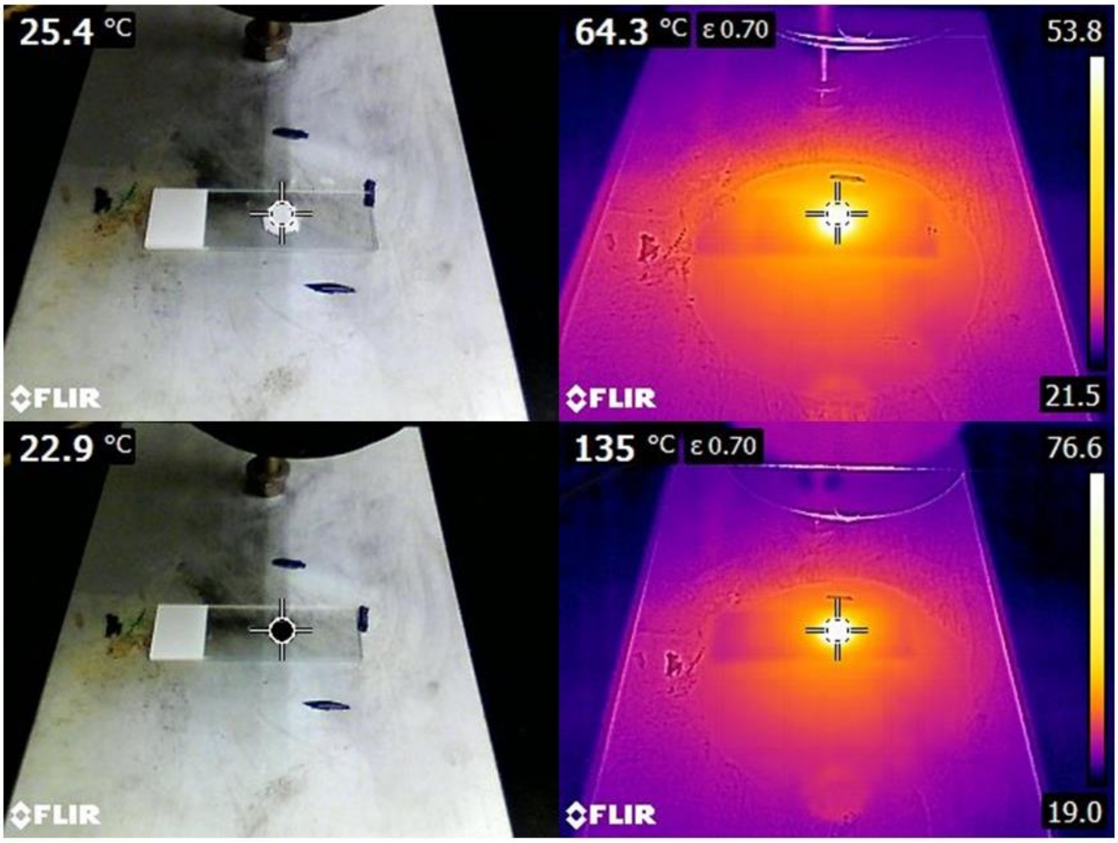

Figure S10. Digital (left) and IR thermal (right) images of TiO_2 tubes (top) and TiN tubes (bottom) under illumination.

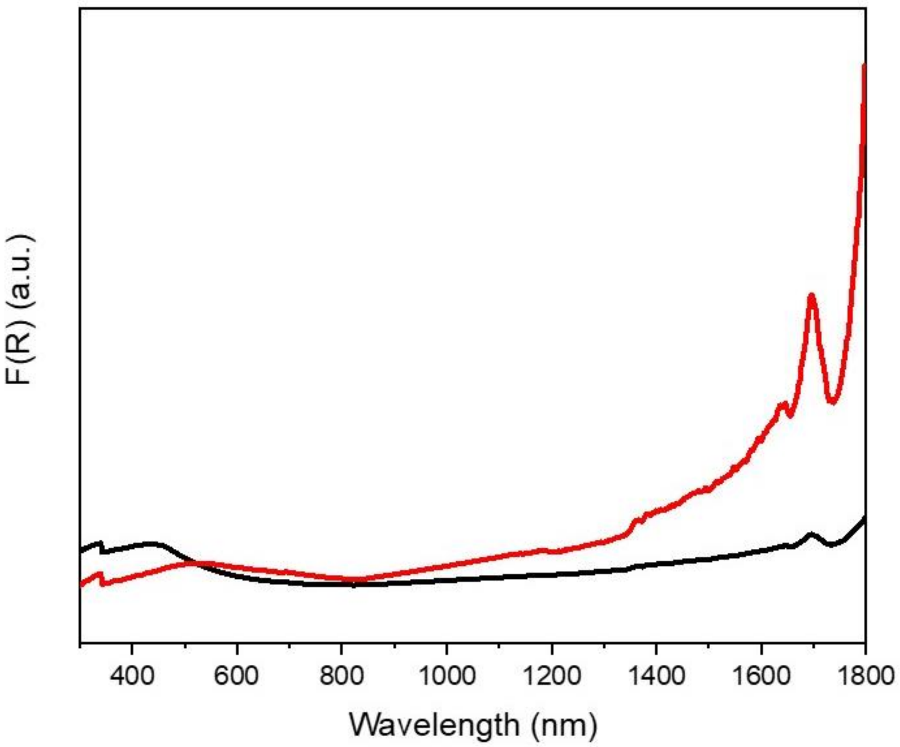

Figure S11. Visible-NIR diffuse-reflectance spectra of commercial TiN (black) and TiN tubes (red).

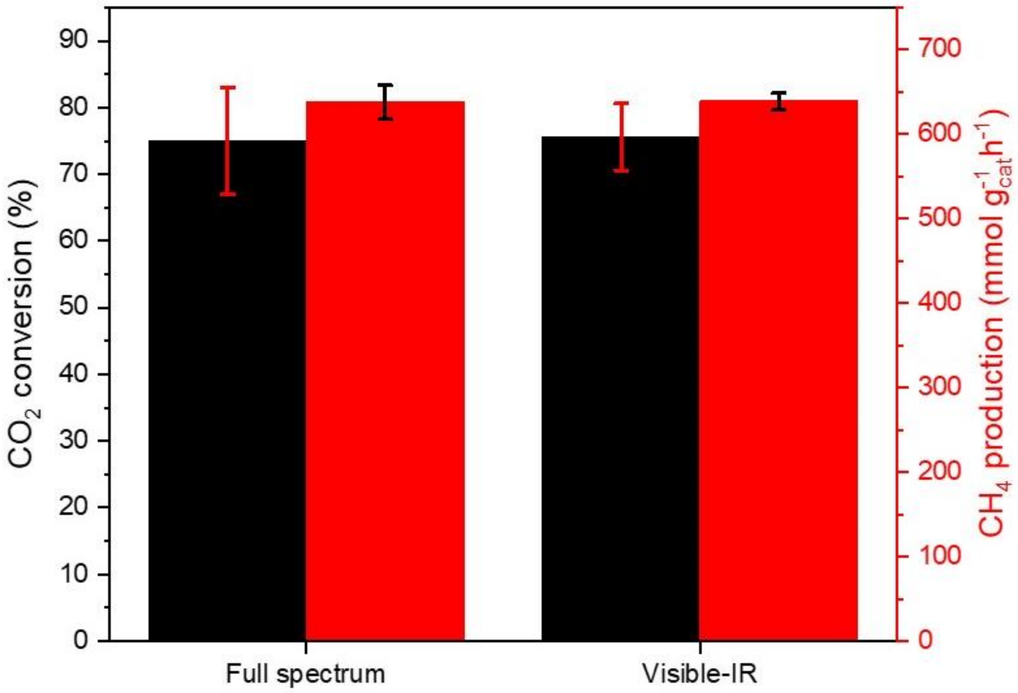

Figure S12. CO_2 conversion (black bars) and CH_4 production rate (red bars) of Ru(2)-TiN tubes under full spectrum and using a UV cut-off filter (λ>420 nm) both under constant power density. Measurements were repeated 3 times.

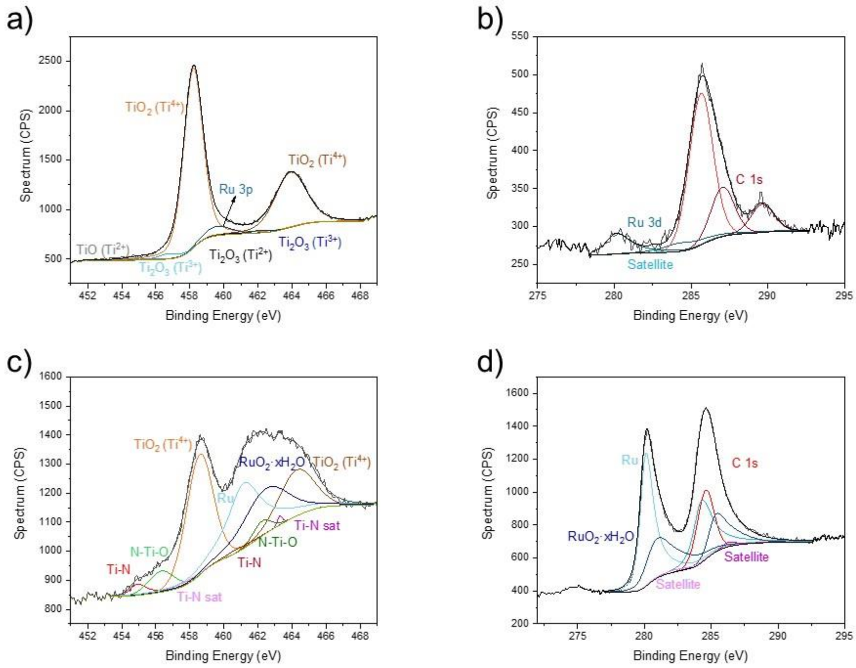

Figure S13. XPS analysis of (**a,c**) Ti2p and (**b,d**) Ru3d regions of Ru-TiO_2 tubes (up) and Ru-TiN commercial (down).

Table S1. Irradiation source, reaction conditions and CH_4 production rate of different high-performance photo-thermal catalysts for the methanation reaction.

Entry	Catalyst	Irradiation Source	Reaction Conditions	CH_4 Production Rate
1	Ru/Al_2O_3 [36]	Xe lamp<br>n.a.	H_2/CO_2 (4:1) Ambient pressure<br>T_{max} = 360 °C	119.8 mmol g_{cat}^{-1} h^{-1}
2	Ru/Si [37]	Xe lamp<br>3.2 kW m^{-2}	H_2/CO_2 (4:1) 45 psi<br>150 °C	0.14 mmol g_{Ru}^{-1} h^{-1}
3	Ru/Si nanowires [37]	Xe lamp<br>3.2 kW m^{-2}	H_2/CO_2 (4:1) 45 psi<br>150 °C	0.99 mmol g_{Ru}^{-1} h^{-1}
4	RuO_2/$SrTiO_3$ [38]	Xe lamp<br>1.3 kW m^{-2}	H_2/CO_2 (4:1) 1.3 bar<br>150 °C	14.6 mmol g_{cat}^{-1} h^{-1}
5	Ru/SiO_2 [39]	Xe lamp<br>0.63 kW m^{-2}	0.5 vol% CO_2/N_2 (50 sccm) and<br>H_2 (1.5 sccm)<br>Ambient pressure<br>300 °C	693.8 mmol g_{Ru}^{-1} h^{-1}
6	Ru@$Ni_2V_2O_7$ [40]	Xe lamp<br>20 kW m^{-2}	H_2/CO_2 (4:1) Ambient pressure<br>T_{max} = 350 °C	114.9 mmol g_{cat}^{-1} h^{-1}
7	Ni/$BaTiO_3$ [10]	Xe lamp<br>3 kW m^{-2}	H_2/CO_2 (4:1) 5 bar<br>T_{max} = 374 °C	103.7 mmol g_{cat}^{-1} h^{-1}
8	Ni@C [42]	Xe lamp<br>43 kW m^{-2}	H_2/CO_2 (4:1) 5 bar<br>T_{max} = 272 °C	488.0 mmol g_{cat}^{-1} h^{-1}
9	Ni/TiO_2 [43]	IR lamp<br>15 kW m^{-2}	H_2/CO_2/He (1:4:5) Ambient pressure<br>T_{max} = 390 °C	463.9 mmol g_{cat}^{-1} h^{-1}
**This work**	**Ru-TiN tubes**	**Xe lamp**<br>**41 kW m^{-2}**	H_2/CO_2 (4:1) 5 bar<br>T_{max} = 287 °C	**1215.8 mmol g_{cat}^{-1} h^{-1}**

References

10. Mateo, D.; Morlanes, N.; Maity, P.; Shterk, G.; Mohammed, O.F.; Gascon, J.; Efficient Visible-Light Driven Photothermal Conversion of CO_2 to Methane by Nickel Nanoparticles Supported on Barium Titanate. *Adv. Funct. Mater.* **2021**, *31*, 2008244.

36. Meng, X.; Wang, T.; Liu, L.; Ouyang, S.; Li, P.; Hu, H.; Kako, T.; Iwai, H.; Tanaka, A.; Ye, J. Photothermal Conversion of CO_2 into CH_4 with H_2 over Group VIII Nanocatalysts: An Alternative Approach for Solar Fuel Production. *Angew. Chem. Int. Ed.* **2014**, *53*, 11478–11482.

37. O'Brien, P.G.; Sandhel, A.; Wood, T.E.; Jelle, A.A.; Hoch, L.B.; Perovi, D.D.; Mims, C.A.; Ozin, G.A. Photomethanation of Gaseous CO_2 over Ru/Silicon Nanowire Catalysts with Visible and Near-Infrared Photons. *Adv. Sci.* **2014**, *1*, 1400001.

38. Mateo, D.; Albero, J.; Garcia, H. Titanium-Perovskite-Supported RuO_2 Nanoparticles for Photocatalytic CO_2 Methanation. *Joule* **2019**, *3*, 1949–1962.

39. Kim, C.; Hyeon, S.; Lee, J.; Kim, W.D.; Lee, D.C.; Kim, J.; Lee, H. Energy-efficient CO_2 hydrogenation with fast response using photoexcitation of CO_2 adsorbed on metal catalysts. *Nat. Commun.* **2018**, *9*, 1–8.

40. Chen, Y.; Zhang, Y.; Fan, G.; Song, L.; Jia, G.; Huang, H.; Ouyang, S.; Ye, J.; Li, Z.; Zou, Z. Cooperative catalysis coupling photo-/photothermal effect to drive Sabatier reaction with unprecedented conversion and selectivity. *Joule* **2021**, *5*, 3235–3251.

41. Khan, I.S.; Mateo, D.; Shterk, G.; Shoinkhorova, T.; Poloneeva, D.; Garzón-Tovar, L.; Gascon, J. Frontispiece: An Efficient Metal–Organic Framework-Derived Nickel Catalyst for the Light Driven Methanation of CO_2. *Angew. Chem. Int. Ed.* **2021**, *60*, 26476–26482.

42. Li, Q.; Gao, Y.; Zhang, M.; Gao, H.; Chen, J.; Jia, H. Efficient infrared-light-driven photothermal CO_2 reduction over MOF-derived defective Ni/TiO_2. *Appl. Catal. B Environ.* **2022**, *303*, 120905.
